# Outcomes Evaluated in Controlled Clinical Trials on the Management of COVID-19: A Methodological Systematic Review

**DOI:** 10.3390/life10120350

**Published:** 2020-12-15

**Authors:** Alexander G. Mathioudakis, Markus Fally, Rola Hashad, Ahmed Kouta, Ali Sina Hadi, Sean Blandin Knight, Nawar Diar Bakerly, Dave Singh, Paula R. Williamson, Tim Felton, Jørgen Vestbo

**Affiliations:** 1 Division of Infection, Immunity and Respiratory Medicine, School of Biological Sciences, The University of Manchester, Manchester Academic Health Science Centre, Manchester M23 9LT, UK; alexander.mathioudakis@manchester.ac.uk (A.G.M.); rola.hashad@manchester.ac.uk (R.H.); akouta28@gmail.com (A.K.); sean.b.knight@gmail.com (S.B.K.); dsingh@meu.org.uk (D.S.); Tim.Felton@manchester.ac.uk (T.F.); 2 North West Lung Centre, Wythenshawe Hospital, Manchester University NHS Foundation Trust, Manchester M23 9LT, UK; 3 Department of Internal Medicine, Section for Pulmonary Diseases, Herlev Gentofte Hospital, 2900 Hellerup, Denmark; markus.fally@regionh.dk; 4 Department of Medical Microbiology and Immunology, Faculty of Medicine, Alexandria University, Alexandria 21131, Egypt; 5 Department of Respiratory Medicine, Salford Royal Infirmary NHS Foundation Trust, Manchester M6 8HD, UK; alisina.hadi91@gmail.com (A.S.H.); Nawar.Bakerly@srft.nhs.uk (N.D.B.); 6 School of Healthcare Sciences, Manchester Metropolitan University, Manchester M15 6BH, UK; 7 School of Healthcare Sciences, Medicines Evaluation Unit, Manchester M23 9QZ, UK; 8 MRC/NIHR Trials Methodology Research Partnership, Department of Health Data Science, University of Liverpool, Liverpool L69 3BX, UK; P.R.Williamson@liverpool.ac.uk

**Keywords:** coronavirus disease 2019, COVID-19, outcomes, endpoints, randomized controlled trials, systematic reviews, trial methods

## Abstract

It is crucial that randomized controlled trials (RCTs) on the management of coronavirus disease 2019 (COVID-19) evaluate the outcomes that are critical to patients and clinicians, to facilitate relevance, interpretability, and comparability. This methodological systematic review describes the outcomes evaluated in 415 RCTs on the management of COVID-19, that were registered with ClinicalTrials.gov, by 5 May 2020, and the instruments used to measure these outcomes. Significant heterogeneity was observed in the selection of outcomes and instruments. Mortality, adverse events and treatment success or failure are only evaluated in 64.4%, 48.4% and 43% of the included studies, respectively, while other outcomes are selected less often. Studies focusing on more severe presentations (hospitalized patients or requiring intensive care) most frequently evaluate mortality (72.5%) and adverse events (55.6%), while hospital admission (50.8%) and viral detection/load (55.6%) are most frequently assessed in the community setting. Outcome measurement instruments are poorly reported and heterogeneous. Follow-up does not exceed one month in 64.3% of these earlier trials, and long-term COVID-19 burden is rarely assessed. The methodological issues identified could delay the introduction of potentially life-saving treatments in clinical practice. Our findings demonstrate the need for greater consistency, to enable decision makers to compare and contrast studies.

## 1. Introduction

The emergence of the coronavirus disease 2019 (COVID-19) led to an unprecedented research mobilization aiming to understand the virus and develop effective preventive and therapeutic strategies [1,2]. Characteristically, within ten months, over 60 thousand publications focusing on COVID-19 were indexed in PubMed and almost two thousand interventional studies were registered with the ClinicalTrials.gov database. However, the limited knowledge about the disease and the need for an expeditious response to the unfolding pandemic did not allow, in some cases, for adequate methodological planning and co-ordination. Extensive research duplication (or better multiplication) has been observed, with numerous randomized controlled trials (RCTs) evaluating the same interventions for COVID-19 in parallel [3]. Moreover, standardization is lacking in trial design and could limit comparability. An important source of variability in trial design could arise from the outcomes (endpoints) that are selected for evaluation. Heterogeneity in trial outcomes and omission of outcomes that are critically important to patients and clinicians complicate interpreting, comparing and synthesizing trial results, potentially delaying the introduction of novel, life-saving treatments into clinical practice [4,5]. 

Core outcome sets are developed to address heterogeneity in the selection of outcomes. These are agreed standardized sets of outcomes that should be measured and reported as a minimum in all clinical trials in specific areas of health or health care [6]. The Core Outcome Measures in Effectiveness Trials (COMET) has developed a rigorous methodology for their development, to ensure the most pertinent clinical outcomes are included in core outcome sets [6,7,8]. Core outcomes should be informed by rigorous methodological systematic review [6,7,8]. 

Upon the emergence of COVID-19 pandemic, there was an urgent need for the development of a core outcome set. Within a few months, four sets were developed, using an accelerated process [9,10,11,12]. These were based on methodological systematic reviews of the first registered RCTs, which were limited in number and design, due to the limited knowledge of the nature of COVID-19, at the time. However, in the meantime, our knowledge of the natural history of COVID-19 is expanding rapidly and numerous clinical trials have been registered. In this methodological survey, we describe the outcomes that are tested in RCTs evaluating therapeutic interventions for COVID-19 and the instruments used to measure these outcomes. 

## 2. Materials and Methods

We followed standard methodology recommended by the Core Outcome Measures in Effectiveness Trials (COMET) initiative for conducting methodological systematic reviews of outcomes evaluated in RCTs [6], that was successfully applied in previous, similar methodological surveys [13,14,15,16]. The Preferred Reporting Items for Systematic Reviews and Meta-Analyses Extension for Scoping Reviews (PRISMA-ScR) was used for reporting this systematic review (Table S1).

### 2.1. Study Selection and Data Extraction

Planned, ongoing or completed interventional clinical trials evaluating pharmacological or non-pharmacological interventions for the management of COVID-19 were considered eligible. Phase 1 trials were considered beyond the scope of this manuscript and, thus, excluded. All eligible trials from the U.S. National Library of Medicine clinical trials register (ClinicalTrials.gov, searched on 5 May 2020) were retrieved using standard filters recommended by the library. More specifically, for identifying studies evaluating COVID-19, we used the following terms: COVID-19, SARS-CoV-2, severe acute respiratory syndrome coronavirus 2, 2019-nCoV, 2019 novel coronavirus, and Wuhan coronavirus. Only studies identified as interventional by the submitting researcher were retrieved.

Eligible studies were grouped into phase 2 or later stage trials, and according to the recruitment setting (community, hospital, or intensive care unit). The main methodological characteristics of all eligible studies, including the planned study population, age of the participants, recruitment setting, blinding, interventions, outcomes, funding, sponsoring, and geographic distribution of the participating centers were extracted automatically from the ClinicalTrials.gov extract (.csv), using a script developed in the platform R statistics (version 3.4.3; R Foundation for Statistical Computing, Vienna, Austria). One researcher (amongst MF, RH, ASH, AK) confirmed eligibility, cross-checked pre-extracted data for accuracy, searched for additional reports of the study protocol and extracted additional data, that were not automatically captured. A second researcher (AGM) cross-checked all extracted data for accuracy. Disagreement was resolved through discussion. Extracted data included the projected recruitment sizes, study settings, as well as details on the eligibility criteria and evaluated outcome measures. 

### 2.2. Outcome Grouping and Classification

Descriptions of all outcome measures were extracted verbatim from the study protocols or registry entries. After in-depth assessment of the outcomes evaluated in a random sample of 20 studies, we developed a list of generic outcome categories defined by the treatment effect they aim to capture, rather than the specific measurement instrument. Two authors (amongst MF, RH, ASH, AK) categorized each of the extracted outcomes within the generic outcome categories. New generic outcome categories were developed as needed, in cases where the evaluated outcomes did not fit any of the existing categories, based on consensus among the co-authors. The instruments used for the quantification of each outcome were also captured. Disagreement was resolved through discussion with another reviewer (AGM). 

Finally, the generic outcomes were further classified according to the COMET taxonomy [17].

## 3. Results

### 3.1. Description of the Included Studies

**Figure A1:**
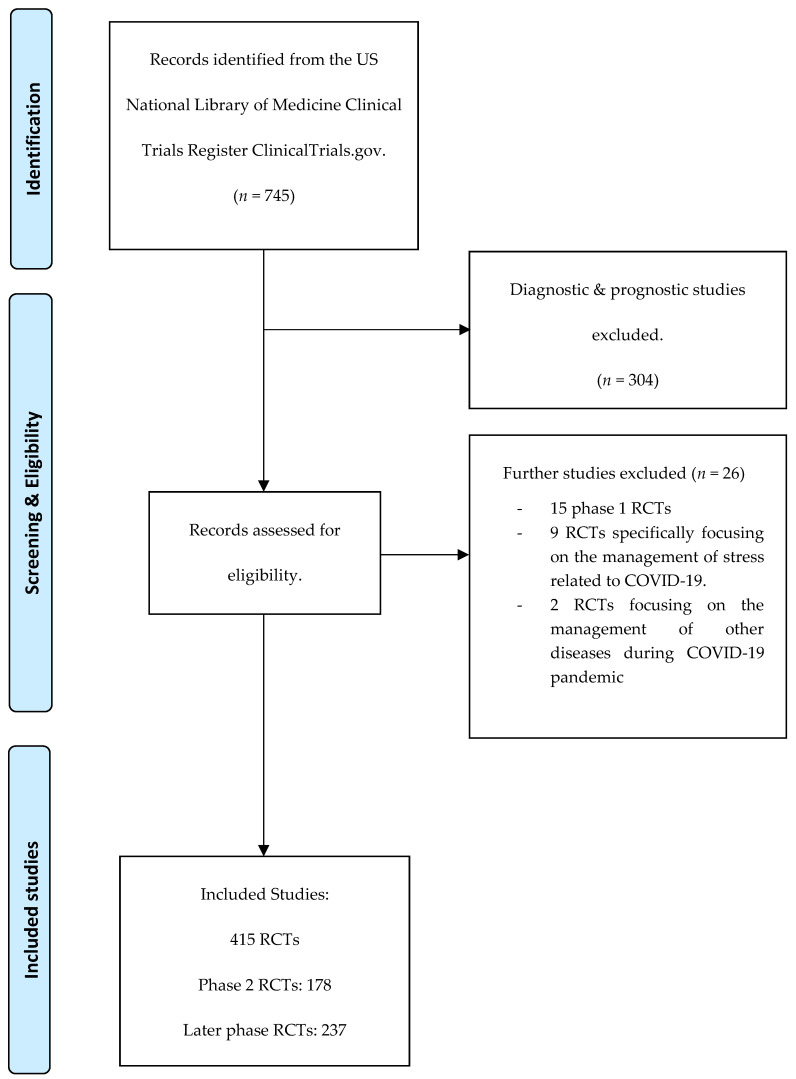
PRISMA flowchart.

Our search retrieved 745 interventional studies. After excluding diagnostic, prognostic, preventive studies, phase 1 trials and those not directly focusing on the management of COVID-19, we selected 415 studies for inclusion in this systematic survey, including 178 phase 2, and 237 later phase RCTs (Figure A1, Table A1). 

Most of the included trials are conducted by academic investigators (75.7%) and only one in four is sponsored by the pharmaceutical industry. The planned recruitment ranges between 7 and 12,000 participants (median: 160, interquartile range [IQR]: 67–400). Most trials include two intervention arms (74.8%), but one in four evaluates more than two, and up to 19 interventions. Moreover, 79.8% of the trials are conducted in a hospital setting, including 6.5% conducted in the intensive care unit (ICU), while 15.2% are conducted in the community. Descriptions of disease severity are heterogeneous, with the recruitment setting being the most consistent measure of disease. Details on the characteristics of the included studies are available in Table 1. 

**Figure A2:**
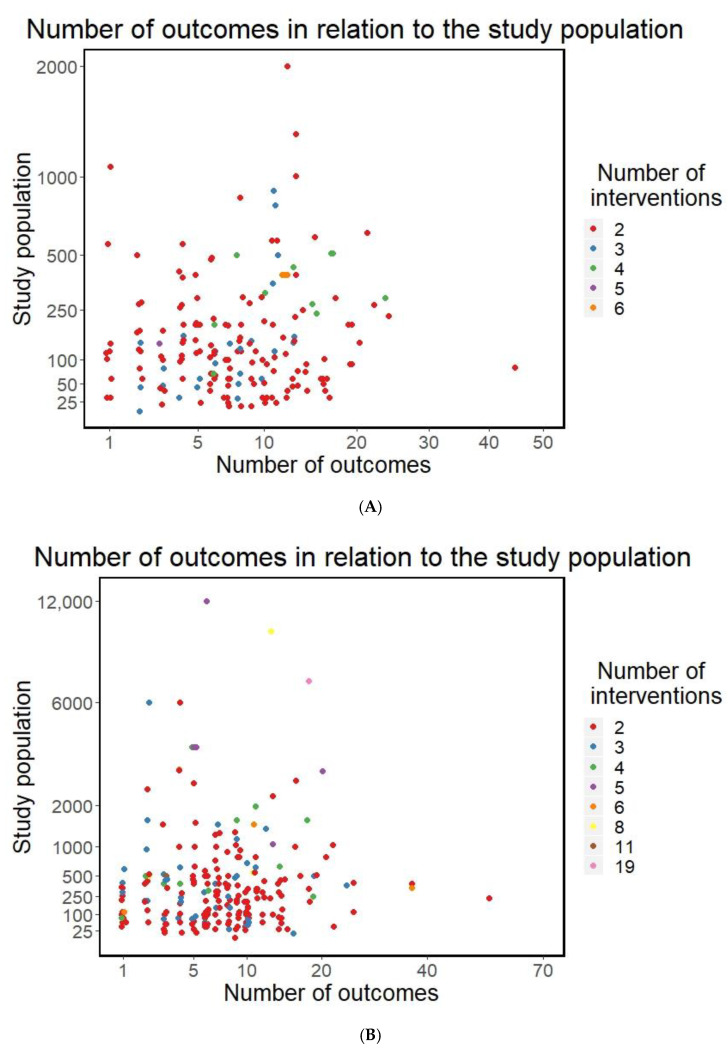
The association between the number of outcomes reported in each RCT and the study population. Having observed that some trials listed numerous inflammatory or other biomarkers as distinct outcomes, for each RCT we have summarized inflammatory biomarkers as a single outcome and other biomarkers as another single outcome. Figure A3 is a non-corrected version of this figure. (**A**) Phase 2 trials, (**B**) Later phase trials.

**Figure A3:**
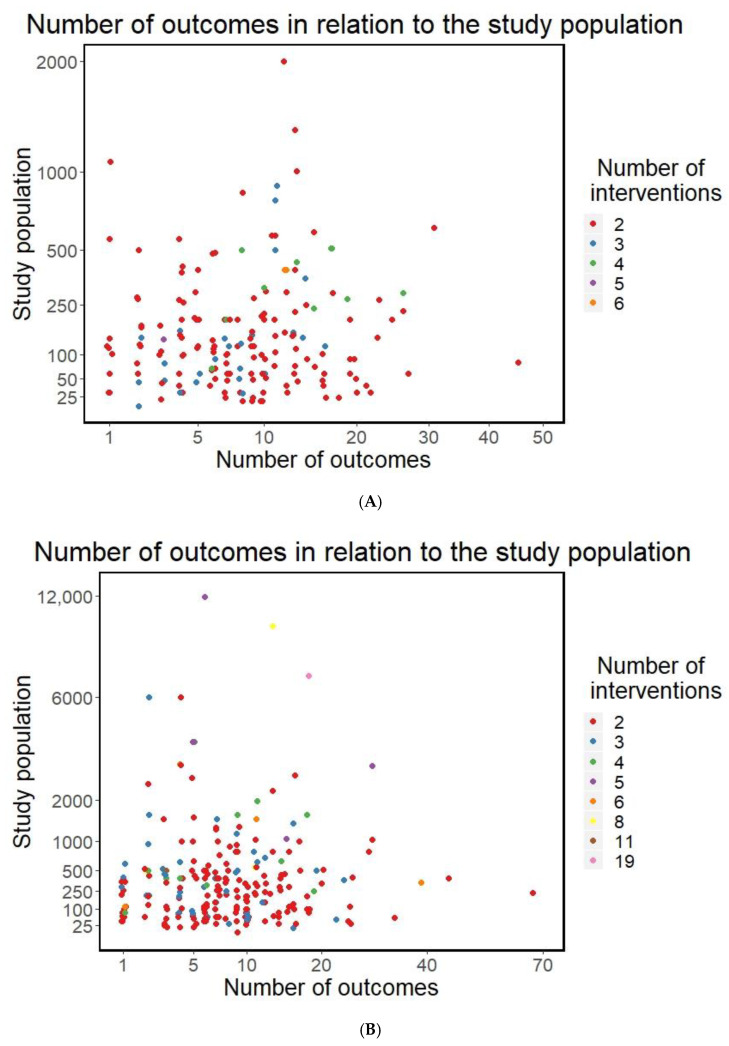
The association between the number of outcomes reported in each RCT and the study population. Non-corrected data. (**A**) Phase 2 trials, (**B**) Later phase trials.

Overall, 3948 unique outcomes are evaluated in the included studies, including 1691 from phase 2 trials and 2257 from later phase trials. We identified 25 generic outcome categories (Table 2). Similar number of outcomes are evaluated in phase 2 (median: 8.5, IQR: 5–13) and later phase (median: 7, IQR: 4–11) trials (Figure A2 and Figure A3). Mortality and adverse events, the most frequently assessed outcomes, are only assessed in 64.6% and 48.4% of all trials, respectively. All remaining outcomes are evaluated in less than half of the trials, highlighting an important heterogeneity in outcomes selection (Table 3 and Table 4). Treatment success or failure is only evaluated in 41.6% of phase 2 trials and 44.1% of the later phase trials. Interestingly, the frequency that different outcomes are evaluated as outcomes or as primary outcomes are very similar for phase 2 and later phase trials.

The most frequently reported outcomes among studies conducted in a community setting (thus recruiting less severely ill patients), were viral detection or load (55.6%), need for hospital admission (50.8%), and symptoms (49.2%). In contrast, the most frequently evaluated outcomes in studies recruiting patients with more severe COVID-19, were mortality and adverse events, which were evaluated in 71.6%, and 50.3% of studies recruiting hospitalized patients, and in 88.9% and 66.7% of those recruiting critically ill patients, respectively. 

### 3.2. Outcome Measurement Instruments

#### 3.2.1. Mortality/Survival (Assessed by 284 Outcomes)

All-cause mortality is evaluated in all but six trials measuring mortality. When mortality was not further described, we presumed it referred to all-cause mortality. Time to death is assessed in 16 trials, and cause-specific mortality in six, mainly focusing on SARS-CoV2 mortality, but also including mortality due to pulmonary or cardiovascular complications.

#### 3.2.2. Clinical Outcomes

1. (Time to) Treatment success or treatment failure: Treatment success or the time to treatment success was evaluated by 220 outcomes. Ordinal scales describing different levels of COVID-19 severity are used for assessing treatment success in 113 (51.4%) of these outcomes. Most scales are very similar to the most frequently used WHO scale, which is a 9-point ordinal scale (from 0 to 8), with each point describing a worse clinical status (Table 5) [18]. Treatment success is defined as an improvement in ordinal scales such as the WHO clinical progression scale by 2 points or 1 point in 57.5% and 24.8% of all outcomes using the scale to evaluate treatment success, while in the remaining outcomes, no specific threshold is provided. Complete resolution of the symptoms and signs of COVID-19 (clinical recovery) is used as a measure of treatment success in 51/220 (23.2%) outcomes and clinical improvement in 38/220 (17.3%) outcomes. The definition of complete resolution varies. Often, no further information is provided. In the remaining cases, it is defined as a composite outcome including several of the following components: complete resolution of breathlessness, tachypnoea, hypoxia, desaturation, cough, anosmia, myalgia, fever, or of oxygen requirements; a negative COVID-19 PCR; hospital discharge; or radiological resolution. A definition of clinical improvement as an outcome is also frequently lacking. In the remaining cases, it is defined as an improvement in several of the previously listed components. Improvement is either based on prespecified thresholds, or on a subjective clinicians’ judgement. Finally, 14 outcomes (6.4%), use specific thresholds (0, ≤2 or ≤4) of the National Early Warning Score (NEWS or NEWS-2) to define treatment success. 

Treatment failure, or time to treatment failure is evaluated by 76 outcomes. In most cases (40/76, 52.6%), treatment failure is defined as a composite outcome consisting of several components with clear thresholds, such as: death, need for ICU admission, need for invasive ventilation, need for other organ support (e.g., vasopressors or renal replacement therapy), need for non-invasive ventilation (NIV), need for supplemental oxygen, a deterioration in oxygenation, need for hospital admission or re-admission or emergency visit, ventricular tachyarrhythmia. Ordinal clinical severity scales such as the WHO scale are used to define treatment failure in 16/76 (21.1%) outcomes, while the need for rescue therapy is used in 9/76 (11.8%) outcomes. The remaining 11 (14.5%) outcomes do not provide specific criteria and/or state treatment failure will be based on the clinician’s judgement of deterioration in the clinical condition of the patient. 

2. Severity scores: Standardized scores are used to evaluate disease severity and progression in 277 outcomes. Ordinal disease severity scales (such as the WHO scale) are the most frequently used scores (144/277 outcomes, 51.2%), followed by the Sequential Organ Failure Assessment (SOFA) Score [19], a validated score for describing the severity of organ dysfunction (54/277 outcomes, 19.5%), and the NEWS score [20]. Acute Physiology and Chronic Health Evaluation II (APACHE II, 5/277), clinical sign score (5/277), Pneumonia Severity Index (PSI, 3/277), BRESCIA-COVID, Murray score, Sepsis Induced Coagulopathy, Small Identification Test, SMART-COP score, and the Vienna Vaccine Safety Initiative (ViVI) disease severity score are used less often.

3. Symptoms: 188 outcomes focus on symptoms, which are either assessed using visual analogue scales, or validated instruments. Composite scores evaluating several symptoms, including breathlessness, cough, sputum production, pyrexia, anosmia, myalgia, headache, or gastrointestinal symptoms are used in 40 outcomes (21.3%). Four composite outcomes specifically assess respiratory symptoms (2.2%). Each of the remaining outcomes focus on a single symptom. These include fever (72/188, 38.3%), breathlessness (18, 9.6%), cough (12, 6.4%), and less often anxiety, depressive symptoms, anosmia, cognitive dysfunction, nausea, insomnia, or fatigue. In this category we also included the assessment of heart rate (8, 4.3%) or blood pressure (5, 2.7%).

#### 3.2.3. Physiological Outcomes

1. Oxygenation (evaluated by 215 outcomes): Oxygenation is evaluated using the partial pressure of oxygen (PaO2), fraction of inspired oxygen (FiO2), oxygen saturation (SatO2), or respiratory rate. Oxygenation is often measured as the PaO2 or SatO2 corrected for FiO2 (95/215, 44.2%). In this category we also included measurements of the partial pressure of carbon dioxide (PaCO2) and pH, which are only rarely evaluated as outcomes. 

2. Pulmonary function and physiology (28 outcomes): There is significant heterogeneity in this domain, with different outcomes evaluating peak flow rate, forced vital capacity (FVC), the ratio of forced expiratory volume in 1 second (FEV1) to FVC, vital capacity, diffusing capacity, lung compliance, and respiratory muscle function. 

3. Viral detection and load (235 outcomes): The vast majority assess virologic clearance by a specific timepoint, or the time until virologic clearance. A small number of outcomes track changes in viral load over time, or differences in the viral detection and load when using different samples (nasal, nasopharyngeal, oropharyngeal swabs or sputum).

4. Viral antibodies: The development of antibodies against SARS-CoV2 is assessed in 31 outcomes. Evaluation of specific antibody types (IgA, IgG, or IgM) is only described in five trials.

5. Radiological outcomes (61 outcomes): Definitions of this outcome are inadequate. In most cases, it is broadly stated that the progression, regression, or resolution of the radiological findings are monitored. Details are only provided in six outcomes, which monitor the extent of the lesion as a proportion of the full lung volume, or perform lung densitometry. Development of fibrosis is evaluated in seven outcomes. Computed tomography (CT) is used in 21 (34.4%) outcomes, a chest X-ray (CXR) in 8 (13.1%), either a CT or a CXR in three, either CT or CXR or lung ultrasound in one and nuclear imaging in one outcome. The imaging modality used is not declared in the remaining 28 (45.9%) outcomes.

6. Inflammatory biomarkers (321 outcomes, each describing either a single or multiple biomarkers): The most frequently evaluated biomarkers are the total white cell count, neutrophils, lymphocytes, eosinophils, monocytes, c-reactive protein, interleukins 1, 6, and 8, followed by other interleukins, procalcitonin, tumour necrosis factors, complement components, lymphocytes subtypes, immunoglobulins, and other inflammatory biomarkers. 

7. Other biomarkers: 309 outcomes evaluate either a single or multiple non-inflammatory biomarkers. Mostly, these are surrogates for safety or adverse events. The most frequently captured biomarkers are d-dimers, cardiac enzymes, kidney function, liver function, clotting, red blood cells and haemoglobin, followed by a variety of other molecules. 

8. Pharmacokinetics/Pharmacodynamics: Here, we categorized 33 outcomes, mostly evaluating plasma drug concentrations (12/33, 36.4%), but also half-life, maximum/minimum observed concentration, time to reach the maximum/minimum observed concentration, area under the plasma concentration-time curve.

#### 3.2.4. Adverse Events

Adverse events (448 outcomes): 108 (24.1%) outcomes evaluate any adverse event; either their frequency, or participants experiencing at least one adverse event. 80 (17.9%) outcomes specifically assess serious adverse events, as defined by the Common Terminology Criteria for Adverse Events (CTCAE). Nineteen (4.2%) outcomes focused on drug reactions, 14 (3.1%) on grade 3 or 4 adverse events, as defined by the CTCAE, and 22 (4.9%) assessed the rate of study drugs discontinuation due to adverse events or due to any reason. The remaining outcomes focused on specific adverse events, mostly cardiac (38, 10.3%), secondary infections (37, 10.0%), thrombotic or bleeding events (29, 8.1%), or local administration reactions (13, 3.6%)

#### 3.2.5. Life Impact (13 Outcomes)

The EuroQol 5 Dimensions (EQ-5D) is used in four outcomes, followed by the Research and Development Corporation’s (RAND) 36-Item Health Survey (SF-36), which is used in three outcomes. Other instruments include the WHO Disability Assessment Schedule (WHODAS 2.0), the Control, Autonomy and Pleasure (CASP-19), and the Nottingham Health Profile.

#### 3.2.6. Resources Use

1. Need for a (higher) level of care (352 outcomes): Need for hospital admission is evaluated by 68 outcomes (19.3%), need for hospital re-admission by 9 (2.6%), need for intensive care admission by 82 (23.4%), need for invasive ventilation by 167 (47.4%), and need for extracorporeal membrane oxygenation (ECMO) by 26 (7.4%; merged with the outcome need for ventilation in the tables). In studies conducted in the hospital setting, need for hospital admission at a specific follow-up timepoint, refers to the proportion of patients who remain inpatients at that timepoint. Similarly, for studies conducted in the ICU, and the need for ICU admission. 

In this category, we also included composite outcomes consisting of one of the above outcomes and mortality (e.g., need for ICU admission or death), as these composite outcomes focus on the need for a higher level of care, while death is added to account for patients who decease before accessing the higher level of care, or those who are not eligible for higher level of care due to their baseline clinical status. Such approaches could be crucial to account for bias, especially in situations such as the COVID-19 pandemic, when hospitals and ICUs are over-burdened and not infrequently unable to accommodate a significant proportion of the patients, leading to the introduction of stricter criteria for triaging patients. Moreover, some outcomes in this category also evaluate time-to-higher level of care (e.g., time-to-hospital admission). 

2. Duration of stay in a specific level of care (469 outcomes): Of those, 206 (43.9%) focus on the length of hospital stay, 96 (20.5%) on the length of ICU stay, and 167 (35.6%) on the duration of invasive ventilation. Delays in discharging patients who are medically optimized due to social or other reasons could introduce bias in the outcome length of hospital stay. To account for this issue, 11 outcomes are defined as the time to discharge or to a NEWS ≤2, maintained for 24 h and another outcome as the time until participants are deemed medically optimized for discharge by a clinician.

3. Need for supplemental oxygen or NIV: This category includes 105 outcomes evaluating the need for supplemental oxygen or NIV in any setting. Most evaluate the need for supplemental oxygen administration at specific follow-up timepoints; 34 (32.4%) outcomes assess the need for NIV (including continuous positive airway pressure [CPAP] or bilevel positive airway pressure [BiPAP]), and 21 (20.0%) the need for high-flow oxygen. One outcome evaluates the need for domiciliary oxygen after hospital discharge. 

4. Duration of supplemental oxygen or NIV (95 outcomes): Twelve (12.6%) evaluate the duration of NIV, and seven (7.4%) evaluate the duration of high-flow oxygen. 

5. Need for other organ support (other than invasive ventilation, 44 outcomes): 26 (59.1%) outcomes focus on the need for vasopressors, and 18 (40.9%) for renal replacement therapy. 

6. Other outcomes: Here, we grouped 145 outcomes that could not be categorized in the previous categories and were evaluated in <10 RCTs each. Need for concurrent treatments is assessed in 22 outcomes, including 7 that specifically focus on the administration of antibiotics. Exercise capacity is assessed by 13 outcomes (mostly using the 6-minutes walking test), COVID-19 transmission by 9, resource requirements, and costs by 8 outcomes. Other outcomes include the use of prone positioning, ability to perform activities of daily living, incidence, and progression of cytokine storm syndrome, resilience, lost workdays, and discharge destinations. 

### 3.3. Study Follow-Up

**Figure 1:**
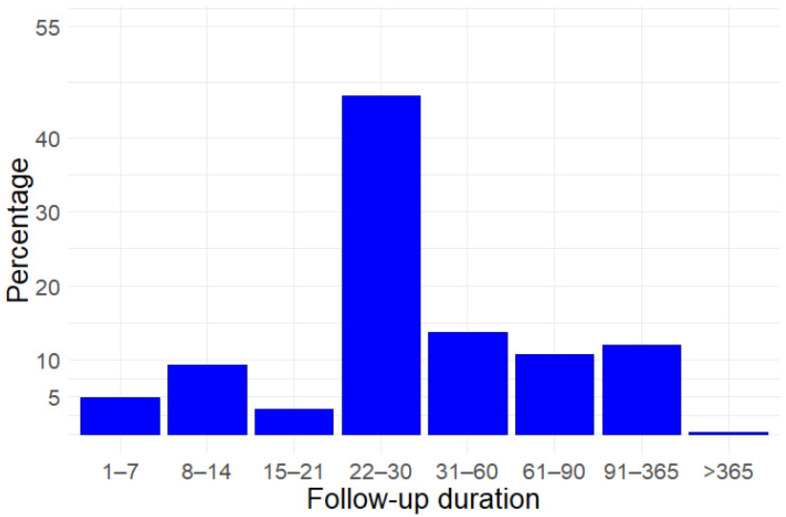
Duration of follow-up in the included studies.

**Figure 2:**
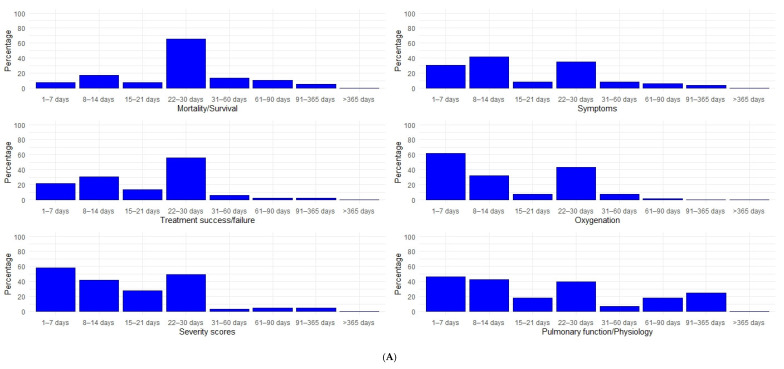

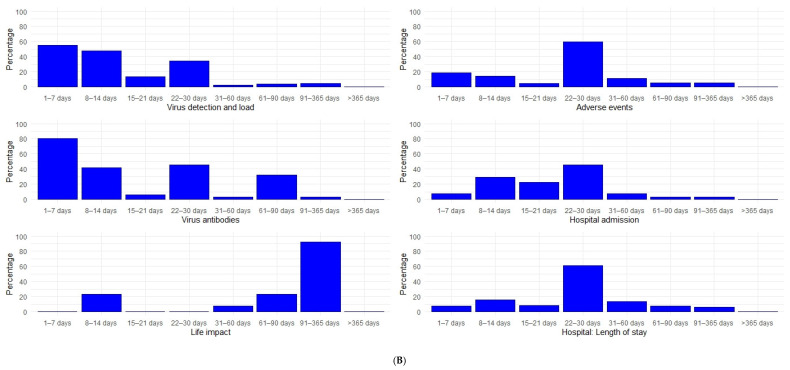

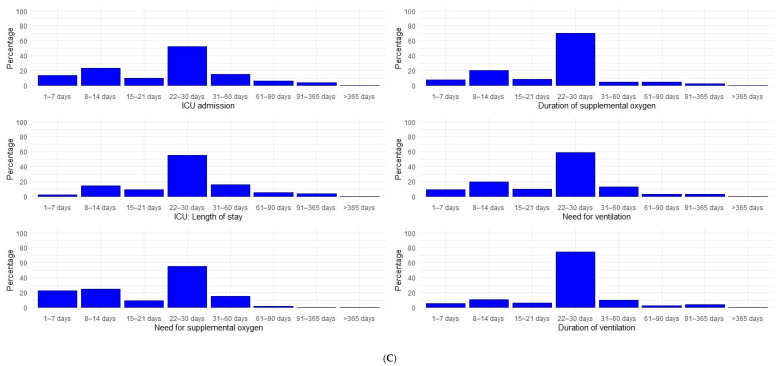
Planned follow-up timepoints for the most frequently evaluated outcomes. All timepoints described in each of the included trials were included in this figure. Presented as a percentage of the outcomes of the same category. (**A**) Mortality/Survival, Treatment success/failure, Severity scores, Symptoms, Oxygenation, Pulmonary function/Physiology, (**B**) Virus detection and load, Virus antibodies, Life impact, Adverse events, Hospital admission, Hospital: Length of stay, (**C**) ICU admission, ICU: Length of stay, Need for supplemental oxygen, Duration of supplemental oxygen, Need for ventilation, Duration of ventilation.

**Figure A4:**
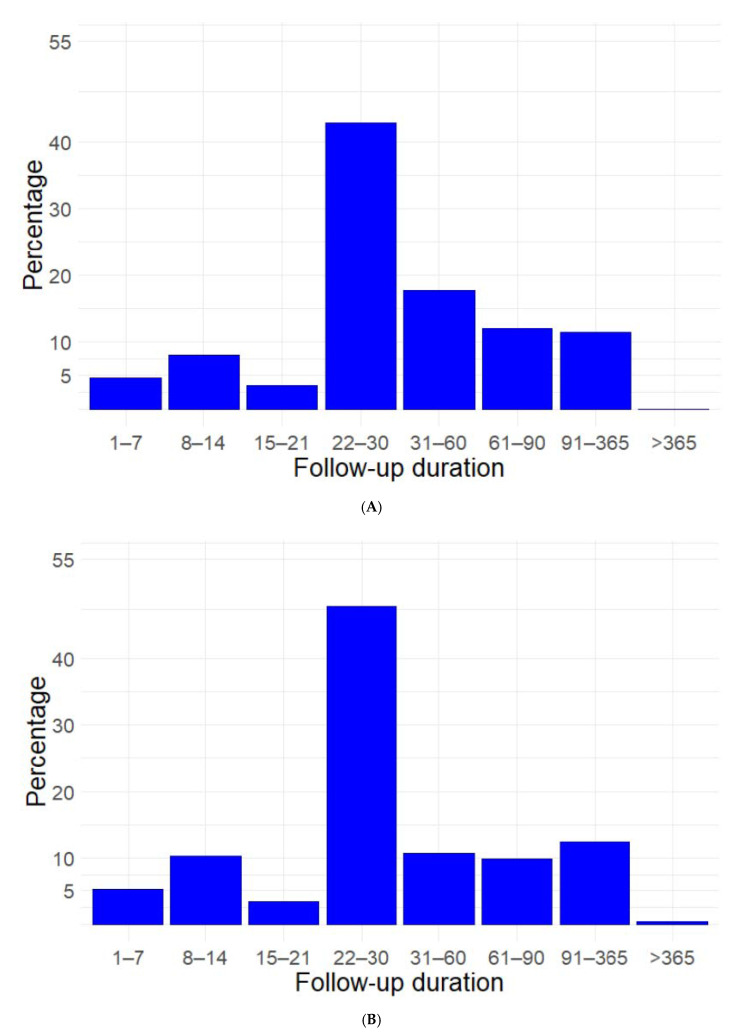
Duration of follow-up in the included studies. (**A**) Phase 2 trials, (**B**) Later phase trials.

**Figure A5:**
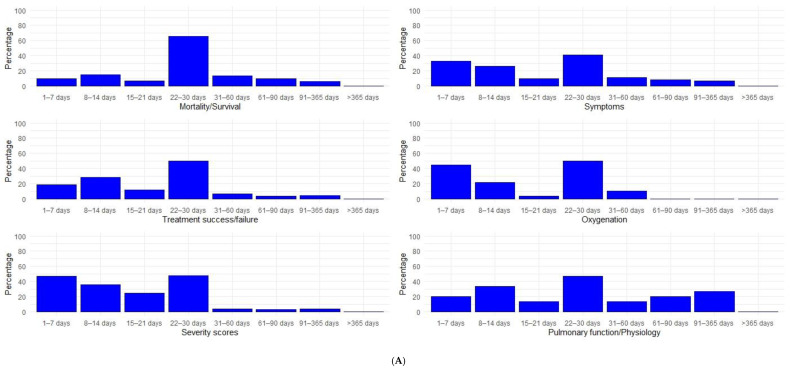

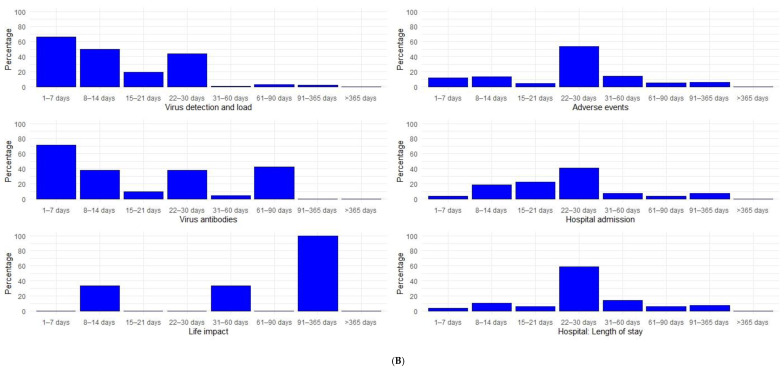

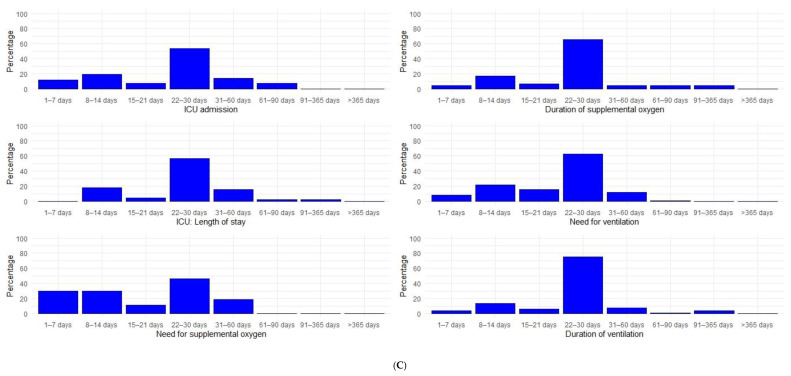

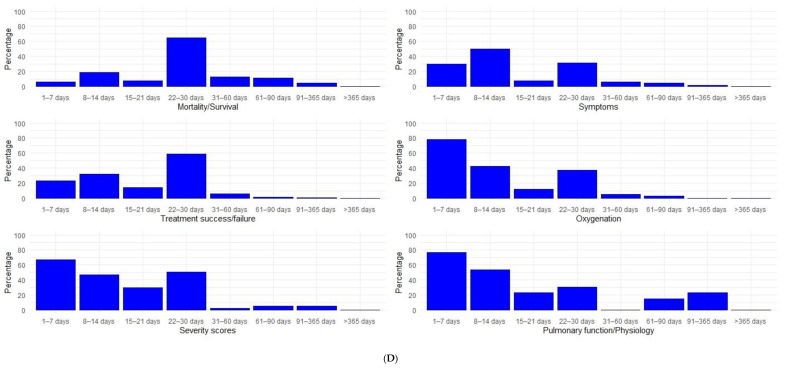

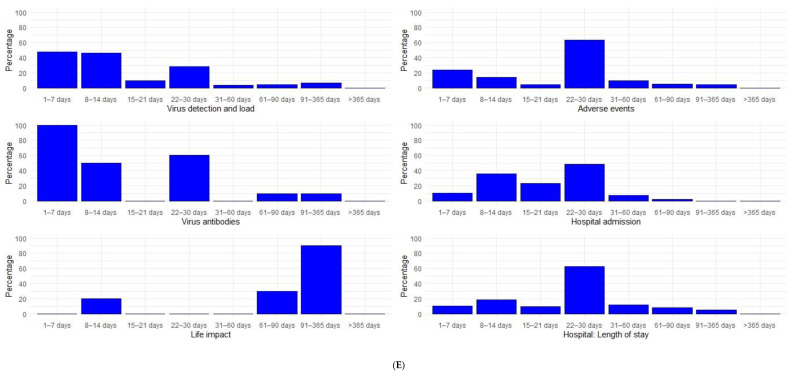

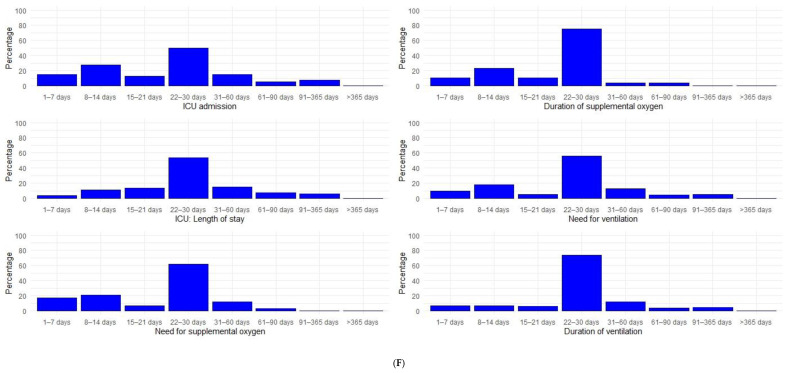
Selected follow-up timepoints for the most frequently evaluated outcomes. All evaluation timepoints described in each of the included trials were included in this figure. Presented as a percentage of the outcomes of the same category. (**A**–**C**) Phase 2 trials, (**D**–**F**) Later phase trials.

Planned follow-up for all included studies varies from less than a week, to over a year (Figure 1, Figure A4). However, in most cases, it does not exceed one month (263/415 63.4%). Follow-up exceeds four months only in 50 (12.0%) studies and one year only in one. Follow-up plans do not differ between phase 2 and later phase trials, where they are limited to one month or less in 105/178 (59.0%) and in 158/237 (66.7%) trials, respectively. Longer-term follow-up, exceeding 4 months, is planned for 163 outcomes (Figure 2, Figure A5), evaluating mortality (16 outcomes), adverse events (15), life impact (12), severity scores (12), length of hospital stay (11), viral detection and load (11), inflammatory biomarkers (7), pulmonary function/physiology (6), need for ventilation (5), and duration of ventilation (5).

## 4. Discussion

In this methodological survey, we analysed the outcomes and outcome measurement instruments used in 415 RCTs evaluating therapeutic interventions for COVID-19. We identified a remarkable heterogeneity in the selection of outcomes, that is not unexpected given that these trials were designed within a few months from the emergence of the new coronavirus strain. More specifically, only 64.6% and 48.4% of the studies evaluate mortality and adverse events, respectively, while each of the remaining outcomes is assessed by markedly less than half of the studies. 

Variability was also observed in the choice of instruments used to measure different outcomes. Ordinal clinical severity scales were consistently used across the included studies to assess treatment success or failure and disease severity. Given the acute nature of COVID-19, and significant changes in the clinical status of patients in the course of the disease, such scales can effectively capture disease progression, especially in more severe presentations. Most of these scales follow the structure of the WHO scale, removing scale points for simplicity. Despite sharing a similar structure, these scales group patients differently, limiting interpretability and comparability. The WHO recently introduced a revised 11-point Scale, with increased granularity, and it would be advisable for all studies to align relevant outcomes with this revised scale, to improve interpretability and comparability [12]. To evaluate treatment success or failure, most studies used a 2-point change in the ordinal scale as a threshold, that corresponds to a significant change in the clinical status of the patient and this seems appropriate. 

Our study revealed a lack of focus on the long-term sequelae of SARS-CoV2 infection. The planned study follow-up exceeds four months only in 12% of all studies. Moreover, only 13 trials assess life impact beyond the acute phase, while exercise capacity is assessed by 13 trials, and the ability to perform simple daily activities during convalescence in only four trials. Only seven trials stated an intent to explore the development of pulmonary fibrosis. However, persistent symptoms, such as fatigue or breathlessness, and quality of life deficits are detected in many hospitalized patients, two to three months after discharge [21,22,23]. Moreover, fibrotic changes are detected in about one in three survivors of a hospitalization for COVID-19 infection [24,25]. However, it should be noted that we evaluated RCTs registered by May 2020 and longer-term follow-up may have been planned for newer studies, in view of the emerging data. 

While this study did not focus on the analytical approaches used for evaluating outcomes, we observed that several studies described specific approaches to account for the bias introduced by mortality as a competing factor for other outcomes, including the duration of hospital stay, ICU stay and the duration of respiratory support. Several methods were described to account for this bias. Some studies stated the duration of hospital or ICU stay will be censored for deceased participants, while others assessed the days that participants are alive and out of hospital or ICU, instead. Homogenization and detailed description of the analytical approaches in the study protocols, along with the outcomes and outcome measurement instruments are crucial for increasing transparency and comparability. Future methodological studies should address analytical approaches.

Four core outcome sets have already been published, with overlapping but not identical selection of components. The WHO Working Group on the Clinical Characterisation and Management of COVID-19 infection recommends the minimal use of three outcomes: mortality, viral burden and non-mortal clinical outcomes evaluated using the WHO clinical progression scale [12]. WHO also highlighted the need for a longer follow-up, of at least 60 days, to capture disease mortality, which is not adopted by most identified trials. Two other groups prioritized specific outcomes and measurement instruments, all of which were captured in our analysis, but were not necessarily the most frequently used [10,11]. The last core outcome set prioritized broader domains to be addressed, rather than specific outcomes [9]. These domains encompass most outcomes identified in this methodological review. The same group also highlighted the need to evaluate the impact of COVID-19 on patient status and life impact in the longer term. Looking across these core outcome sets, a meta-core outcome set (meta-COS) was identified, only including the two domains that were prioritized by all initiatives (mortality and respiratory support), as the most critical, to be evaluated in all future RCTs in hospitalized patients [26]. Both domains recommended by the meta-COS were evaluated in 205 (49.4%) of the included studies. 

In view of the multiple available core outcome sets, the authors of this review believe that outcomes selection for future trials should (i) adhere to the recommendations by the WHO and the meta-COS, and (ii) attempt to address all of the domains proposed by Tong et al., a core outcome set that was informed by consensus of >9000 participants [9]. Undeniably, the objectives of individual trials vary and, accordingly, additional outcomes could be selected to address specific trial objectives. However, evaluating the most pertinent outcomes summarized in the previously mentioned core outcomes could improve the interpretability and comparability of their results. 

Methodological systematic reviews were conducted as part of the development of three core outcome sets. However, these reviews were almost exclusively based on studies conducted in China. Moreover, two of these reviews included approximately 100 RCT protocols [10,11], while the WHO document was informed by 1135 protocols, including both observational and interventional studies [12]. However, the outcomes of RCTs often differ from those selected in observational studies. Our methodological review was based on a globally representative sample of 415 RCTs, it employed more rigorous methodology to assess all outcomes, and it is the first review to evaluate the instruments used to evaluate the different outcomes beyond mortality.

Our study only included clinical trials that were registered until May 2020 and this may be a limitation as trial designs and endpoints may have evolved since then, in view of the emerging knowledge on the nature and outcomes of COVID-19 infection, and the published core outcome sets. Importantly, the study protocols of some of the included RCTs have been amended since then and our methodological systematic review is a snapshot of the RCT designs and plans as of May-August 2020. Moreover, we only evaluated studies registered with the U.S. National Library of Medicine clinical trials register (ClinicalTrials.gov). However, our extensive, globally representative sample of 415 ongoing RCTs was a major strength of our methodological survey and we strongly believe it was sufficient to capture all relevant outcomes and measurement instruments. Characteristically, after extracting data from approximately 25% and 70% of the included trials, we reached saturation with regards to the outcome categories and the outcome measurement instruments, respectively. Therefore, we are confident that we have not missed important outcomes by focusing exclusively on clinicaltrials.gov. Future studies will need to assess the impact of the emerging evidence on the natural history and outcomes of COVID-19 and of the four published core outcome sets and the meta-COS on the selection of outcomes in more recently registered trials. Another limitation of our study is the lack of a prospectively registered protocol. However, we have used rigorous methodology recommended by the COMET Initiative, that we have previously employed in similar methodological systematic reviews [13].

Overall, this methodological survey reveals significant heterogeneity in the outcome categories and measurement instruments selected by trialists in the management of COVID-19 and highlights the need for greater consistency, to enable decision-makers to compare and contrast studies.

## Figures and Tables

**Table 1:** Characteristics of the included studies. * Studies conducted in multiple continents are counted in each participating continent.

Study Characteristics	Phase 2 Trials (*n* = 178)	Later Phase Trials (*n* = 237)
**Number of participants**		
Median (range)	120 (15–2000)	253 (7–12,000)
**Setting**		
Community	25 (14.0%)	38 (16.0%)
Hospital	137 (77.0%)	167 (70.5%)
Community and Hospital	3 (1.7%)	1 (0.4%)
ICU	9 (5.1%)	18 (7.6%)
Other	0 (0.0%)	1 (0.4%)
Unclear	4 (2.2%)	13 (5.5%)
**Continent**		
Africa	5 (2.8%)	21 (8.9%)
Asia	29 (16.3%)	51 (21.5%)
Europe	46 (25.8%)	94 (39.7%)
North America	90 (50.6%)	67 (28.3%)
Oceania	1 (0.6%)	1 (0.4%)
South America	12 (6.7%)	22 (9.3%)
Multiple continents *	6 (3.4%)	15 (6.3%)
Unclear	6 (3.4%)	0 (0.0%)
**Age range**		
**Minimum age**		
Median (range)	18 (3–50)	18 (1–70)
Not reported	2 (1.1%)	0 (0.0%)
**Maximum age**		
Median (range)	80 (50–110)	80 (40–110)
Not reported	115 (64.6%)	157 (66.0%)
**Number of interventions**		
2	139 (78.1%)	172 (72.6%)
3	25 (14.0%)	40 (16.9%)
4	10 (5.6%)	11 (4.6%)
5	1 (0.6%)	5 (2.1%)
6	3 (1.7%)	4 (1.7%)
8	0 (0.0%)	3 (1.3%)
11	0 (0.0%)	1 (0.4%)
19	0 (0.0%)	1 (0.4%)
**Sponsor**		
Academic	124 (69.7%)	190 (80.2%)
Pharmaceutical industry	54 (30.3%)	47 (19.8%)

**Table 2:** Definitions of the generic outcome categories.

Outcome Categories	Definitions
**Mortality/Survival**	Evaluation the survival status.
**Clinical/Physiological**	
Treatment success or treatment failure	A clinical evaluation of whether COVID-19 was successfully treated. Usually a composite endpoint based on one or more of the following: survival, symptoms progression or regression, pyrexia regression, oxygen requirements and/or the requirement for ventilation. We only considered in this category binary outcomes describing criteria either for treatment success or treatment failure. Time-to-treatment success or failure is a measurement instrument that could provide more granular information.
Severity scores	A quantitative evaluation of disease severity. In this category we included outcomes presenting mean/median scores or change from baseline in a validated score. Outcomes describing predefined score thresholds for treatment success or failure were classified in the previous category.
Symptoms	Quantitative or qualitative evaluation of the intensity of one or more symptoms, including but not limited to breathlessness, cough, pyrexia or anosmia.
Oxygenation	Physiological measures of oxygenation, including oxygen saturation (SatO2), the partial pressure of oxygen (PaO2) or carbon dioxide (PaCO2). The need for supplementary oxygen or ventilation were summarized in separate outcome categories.
Pulmonary function and physiology	Measures of pulmonary functions and lung physiology including the forced expiratory volume in 1 second (FEV1), forced vital capacity (FVC), respiratory muscle strength or the lung compliance.
Viral detection and load	Polymerase chain reaction (PCR) to evaluate the presence, persistence and/or load of the severe acute respiratory syndrome coronavirus-2 (SARS-CoV2).
Viral antibodies	Detection of the presence and titres of antibodies against SARS-CoV2.
Radiological outcomes	Radiological progression in chest x-ray (CXR) or computed tomography (CT) of the chest.
Inflammatory biomarkers	The levels and trajectories of any inflammatory biomarkers, including white blood cells count, lymphocytes, neutrophils, eosinophils, monocytes, CD4+ or CD8+ T cell counts, c-reactive protein, interleukins, tumour necrosis factors, or any other inflammatory biomarkers.
Other biomarkers	The levels and trajectories of any other biomarkers, including but not limited to kidney function, liver function, haematocrit, coagulation profile, d-dimers, troponin or the brain natriuretic peptide (BNP).
Pharmacokinetics/pharmacodynamics	Evaluation of the pharmacokinetics and/or pharmacodynamics of the drug interventions (mainly serum levels over time).
**Adverse events**	Adverse events or grade 3 or more severe adverse events, or serious adverse events, according to the Common Toxicity Criteria for Adverse Events (CTCAE). In this category, we also included outcomes evaluating specific adverse events, such as renal failure, liver failure, pulmonary embolism, myocardial infarction or tachyarrhythmias. Treatment discontinuation was also included in this category.
**Life impact**	Quantitative assessment of the general well-being of participants.
**Resource use**	
Need for (higher) level of care	This group of outcomes include the need for (i) hospital admission, (ii) hospital re-admission, (iii) intensive care admission, (iv) invasive ventilation, or need for ECMO. In each category, we also included the composite outcomes consisting of the need for the specific level of care or death. For example: “intensive care admission or death”, as these composite outcomes were developed to account for patients who might have benefitted by the higher level of care but died or patients who were not eligible for the higher level of care due to their baseline clinical status. In studies conducted in the hospital setting, need for hospital admission at a specific follow-up timepoint, refers to the proportion of patients who remain inpatients at that timepoint. Similarly, for studies conducted in the ICU stay and the need for ICU admission.
Duration of stay in a specific level of care	This group of outcomes include length of (i) hospital stay, (ii) intensive care admission, or (iii) mechanical ventilation. The end date was often defined as the last day of stay in a specific level of care, or the last day that the stay was indicated (to account for cases when patients are medically optimized for hospital discharge but remain at hospital for social or other reasons.
Need for supplemental oxygen or NIV	An assessment of the need for supplemental oxygen, the required oxygen flow or modality of delivery (e.g., oxygen, continuous positive airways pressure [CPAP], bilevel positive airway pressure [BiPAP], or high flow nasal oxygen).
Duration of supplemental oxygen or NIV	An evaluation of the duration of supplemental oxygen needs.
Need for other organ support	This category included the need for (a) vasopressors and (b) need for renal replacement therapy.
**Other outcomes**	In this category we summarized outcomes that were reported in less than 10 of all eligible trials. These included changes in activities of daily living, quality of life, pharmacodynamics and pharmacokinetics, drug compliance, feasibility outcomes, use of antibiotics or other drugs, emergency room visits or use of other healthcare resources, the need for prone positioning, need for transfusion and discharge destinations.

**Table 3:** Frequency that outcome measures are reported in randomized controlled trials (RCTs) on the management of coronavirus disease 2019 (COVID-19). RCTs grouped in phase 2 and later phase trials. Outcomes evaluated in <10 RCTs were grouped as “Other outcomes”. Time to treatment success or failure is a measurement instrument of the outcome treatment success or failure. However, it is reported separately here, as it provides more granular information. NIV: Non-invasive ventilation.

Outcome Category	Phase 2 Trials (*n* = 178)		Later Phase Trials (*n* = 237)	
	Any Outcome	Primary Outcome	Any Outcome	Primary Outcome
**Mortality/survival**	115 (64.6%)	24 (13.5%)	153 (64.6%)	32 (13.5%)
**Clinical/physiological outcomes**				
Treatment success or treatment failure	70 (39.3%)	31 (17.4%)	103 (43.5%)	69 (29.1%)
Success	55 (30.9%)	19 (10.7%)	88 (37.1%)	54 (22.8%)
Failure	23 (12.9%)	12 (6.7%)	31 (13.1%)	14 (5.9%)
Subgroup: Time to treatment success or treatment failure	37 (20.2%)	12 (6.7%)	62 (26.2%)	36 (15.2%)
Success	30 (16.9%)	9 (5.1%)	59 (24.9%)	33 (13.9%)
Failure	8 (4.5%)	3 (1.7%)	11 (4.6%)	3 (1.3%)
Severity scores	76 (42.7%)	21 (11.8%)	93 (39.2%)	25 (10.5%)
Symptoms	43 (24.2%)	5 (2.8%)	60 (25.3%)	7 (3.0%)
Oxygenation	63 (35.4%)	22 (12.4%)	72 (30.4%)	23 (9.7%)
Pulmonary function/physiology	12 (6.7%)	3 (1.7%)	9 (3.8%)	1 (0.4%)
Viral detection and load	59 (33.1%)	20 (11.2%)	97 (40.9%)	36 (15.2%)
Viral antibodies	17 (9.6%)	0 (0.0%)	8 (3.4%)	2 (0.8%)
Radiological outcomes	25 (14.0%)	3 (1.7%)	25 (10.5%)	9 (3.8%)
Inflammatory biomarkers	69 (38.8%)	7 (3.9%)	66 (27.8%)	9 (3.8%)
Other biomarkers	47 (26.4%)	4 (2.2%)	51 (21.5%)	2 (0.8%)
Pharmacokinetics/pharmacodynamics	10 (5.6%)	0 (0.0%)	5 (2.1%)	0 (0.0%)
**Adverse events**	95 (53.4%)	18 (10.1%)	121 (51.1%)	8 (3.4%)
**Life impact**	3 (1.7%)	1 (0.6%)	10 (4.2%)	0 (0.0%)
**Resource use**				
Hospital admission	21 (11.8%)	9 (5.1%)	30 (12.7%)	18 (7.6%)
Hospital re-admission	6 (3.4%)	1 (0.6 %)	3 (1.3%)	0 (0.0%)
Length of hospital stay	70 (39.3%)	5 (2.8%)	103 (43.5%)	7 (3.0%)
ICU admission	35 (19.7%)	6 (3.4%)	38 (16.0%)	2 (0.8%)
Length of ICU stay	42 (23.6%)	0 (0.0%)	49 (20.7%)	3 (1.3%)
Need for supplemental oxygen or NIV	31 (17.4%)	12 (6.7%)	44 (18.6%)	3 (1.3%)
Duration of supplemental oxygen or NIV	40 (22.5%)	2 (1.1%)	39 (16.5%)	1 (0.4%)
Need for invasive ventilation	62 (34.8%)	16 (9.0%)	87 (36.7%)	27 (11.4%)
Duration of invasive ventilation	65 (36.5%)	9 (5.1%)	68 (28.7%)	9 (3.8%)
Need for vasopressors	11 (6.2%)	0 (0.0%)	10 (4.2%)	0 (0.0%)
Need for renal replacement therapy	6 (3.4%)	0 (0.0%)	7 (3.0%)	0 (0.0%)
**Other outcomes**	31 (17.4%)	2 (1.1%)	42 (17.7%)	5 (2.1%)

**Table 4:** Frequency that outcome measures are reported in RCTs on the management of COVID-19. RCTs grouped by recruitment setting (community, hospital, intensive care unit (ICU)). Outcomes evaluated in <10 RCTs were grouped as “Other outcomes”. Time to treatment success or failure is a measurement instrument of the outcome treatment success or failure. However, it is reported separately here, as it provides more granular information. NIV: Non-invasive ventilation. * Continued need of hospital/critical care admission, at a specific timepoint.

Outcome Category	Community (*n* = 63)		Hospital (*n* = 304)		ICU (*n* = 27)	
	Any Outcome	Primary Outcome	Any Outcome	Primary Outcome	Any Outcome	Primary Outcome
**Mortality/survival**	19 (30.2%)	3 (4.8%)	216 (71.6%)	44 (14.5%)	24 (88.9%)	8 (29.6%)
**Clinical/Physiological Outcomes**						
Treatment success or treatment failure	25 (39.7%)	15 (23.8%)	140 (46.2%)	81 (26.6%)	2 (7.4%)	0 (0.0%)
Success	16 (25.4%)	7 (11.1%)	121 (39.8%)	63 (20.7%)	1 (3.7%)	0 (0.0%)
Failure	12 (19.0%)	8 (12.7%)	41 (13.5%)	17 (5.6%)	1 (3.7%)	0 (0.0%)
Subgroup: Time to treatment success or treatment failure	12 (19.0%)	5 (7.9%)	83 (27.3%)	40 (13.2%)	0 (0.0%)	0 (0.0%)
Success	7 (11.1%)	3 (4.8%)	79 (26.0%)	37 (12.2%)	0 (0.0%)	0 (0.0%)
Failure	4 (6.3%)	2 (3.2%)	13 (4.3)	3 (0.9%)	0 (0.0%)	0 (0.0%)
Severity scores	16 (25.4%)	5 (7.9%)	136 (44.7%)	40 (13.2%)	12 (44.4%)	1 (3.7%)
Symptoms	31 (49.2%)	4 (6.3%)	61 (20.1%)	7 (2.3%)	2 (7.4%)	0 (0.0%)
Oxygenation	6 (9.5%)	2 (3.2%)	110 (36.2%)	35 (11.5%)	15 (55.6%)	7 (25.9%)
Pulmonary function/physiology	1 (1.6%)	1 (1.6%)	12 (3.9%)	1 (0.3%)	5 (18.6%)	0 (0.0%)
Viral detection and load	35 (55.6%)	18 (28.6%)	107 (35.2%)	34 (11.1%)	7 (25.9%)	0 (0.0%)
Viral Antibodies	4 (6.3%)	0 (0.0%)	19 (6.3%)	2 (0.7%)	1 (3.7%)	0 (0.0%)
Radiological outcomes	4 (6.3%)	3 (4.8%)	40 (13.2%)	8 (2.6%)	3 (11.1%)	0 (0.0%)
Inflammatory biomarkers	6 (9.5%)	1 (1.6%)	114 (37.5%)	14 (4.6%)	11 (40.7%)	1 (3.7%)
Other biomarkers	4 (6.3%)	0 (0.0%)	79 (26.0%)	5 (1.6%)	10 (37.0%)	0 (0.0%)
Pharmacokinetics / Pharmacodynamics	2 (3.2%)	0 (0.0%)	13 (4.3%)	0 (0.0%)	0 (0.0%)	0 (0.0%)
**Adverse events**	25 (39.7%)	3 (4.8%)	166 (54.6%)	21 (6.9%)	18 (66.7%)	2 (7.4%)
**Life Impact**	0 (0.0%)	0 (0.0%)	7 (2.3%)	0 (0.0%)	3 (11.1%)	0 (0.0%)
**Resource Use**						
Hospital admission	32 (50.8%)	21 (33.3%)	15 (4.9%) *	4 (1.3%) *	1 (3.7%) *	0 (0.0%) *
Hospital re-admission	0 (0.0%)	0 (0.0%)	9 (3%)	1 (0.3%)	0 (0.0%)	0 (0.0%)
Length of hospital stay	9 (14.3%)	1 (1.6%)	152 (50%)	11 (36.2%)	10 (37.0%)	1 (3.7%)
ICU admission	8 (12.7%)	0 (0.0%)	61 (20.1%) *	8 (2.6%) *	2 (7.4%) *	0 (0.0%) *
Length of ICU stay	5 (7.9%)	1 (1.6%)	70 (23.0%)	1 (0.3%)	14 (51.9%)	1 (3.7%)
Need for supplemental oxygen or NIV	4 (6.3%)	0 (0.0%)	68 (22.4%)	13 (4.3%)	1 (3.7%)	0 (0.0%)
Duration of supplemental oxygen or NIV	3 (4.8%)	0 (0.0%)	70 (23.0%)	3 (0.9%)	3 (11.1%)	0 (0.0%)
Need for invasive ventilation	7 (11.1%)	2 (3.2%)	130 (42.8%)	34 (11.2%)	6 (22.2%)	4 (14.8%)
Duration of invasive ventilation	5 (7.9%)	1 (1.6%)	106 (34.9%)	10 (3.3%)	19 (70.4%)	7 (25.9%)
Need for vasopressors	0 (0.0%)	0 (0.0%)	18 (5.9%)	0 (0.0%)	2 (7.4%)	0 (0.0%)
Need for renal replacement therapy	0 (0.0%)	0 (0.0%)	10 (3.3%)	0 (0.0%)	3 (11.1%)	0 (0.0%)
**Other outcomes**	13 (20.6%)	3 (4.8%)	44 (14.5%)	5 (1.6%)	8 (29.6%)	0 (0.0%)

**Table 5:** The WHO 9-point ordinal clinical progression scale [18].

Patient State	Descriptor	Score
**Uninfected**	No clinical or virological evidence of infection	**0**
**Ambulatory**	No limitation of activities	**1**
	Limitation of activities	**2**
**Hospitalized,** **Mild disease**	Hospitalized, no oxygen therapy	**3**
	Hospitalized, oxygen therapy by mask or nasal prongs	**4**
**Hospitalized,** **Severe disease**	Non-invasive ventilation or high-flow oxygen	**5**
	Intubation and mechanical ventilation	**6**
	Ventilation and additional organ support (vasopressors, renal replacement therapy, or ECMO)	**7**
**Dead**	Death	**8**

## Supplementary Materials

The following are available online at https://www.mdpi.com/2075-1729/10/12/350/s1, Table S1. Preferred Reporting Items for Systematic reviews and Meta-Analyses extension for Scoping Reviews (PRISMA-ScR) Checklist.

## Appendix A

**Table A1:** Registration numbers of the included studies. N: Planned study population.

NCT Number	N	NCT Number	N	NCT Number	N	NCT Number	N
NCT04336904	100	NCT04346147	165	NCT04324528	30	NCT04393038	1034
NCT04345445	310	NCT04360876	90	NCT04343651	75	NCT04392141	200
NCT04359095	1600	NCT04336332	160	NCT04366908	1008	NCT04405102	48
NCT04347915	60	NCT04357444	30	NCT04349618	200	NCT04385043	400
NCT04333407	3170	NCT04317040	230	NCT04355962	64	NCT04401579	1032
NCT04336462	100	NCT04342897	200	NCT04347239	390	NCT04390594	258
NCT04342689	1500	NCT04365231	50	NCT04323800	487	NCT04385940	64
NCT04376788	15	NCT04346368	20	NCT04321096	580	NCT04405310	80
NCT04333420	130	NCT04371406	2770	NCT04268537	120	NCT04380519	372
NCT04360356	100	NCT04288102	90	NCT04332666	60	NCT04382755	81
NCT04370262	942	NCT04286503	520	NCT04349098	230	NCT04400890	200
NCT04350593	900	NCT04351581	215	NCT04332835	80	NCT04391712	20
NCT04372979	80	NCT04351243	270	NCT04361461	500	NCT04394416	204
NCT04325633	584	NCT04347512	405	NCT04366271	106	NCT04393311	150
NCT04339660	30	NCT04339816	240	NCT04366089	152	NCT04392414	60
NCT04362813	450	NCT04347980	122	NCT04373733	450	NCT04398303	70
NCT04354389	82	NCT04357808	30	NCT04312009	200	NCT04391127	200
NCT04362137	402	NCT04358926	30	NCT04361474	120	NCT04396106	180
NCT04359615	40	NCT04293692	0	NCT04315948	3100	NCT04405843	400
NCT04359316	40	NCT04362176	500	NCT04311177	580	NCT04381052	30
NCT04343768	60	NCT04338828	260	NCT04261426	80	NCT04397562	204
NCT04280705	800	NCT04353180	45	NCT04255017	400	NCT04385264	800
NCT04329832	300	NCT04371952	330	NCT04254874	100	NCT04385264	800
NCT04365257	220	NCT04335305	24	NCT04341935	20	NCT04382586	52
NCT04350671	40	NCT04347538	90	NCT04261270	60	NCT04381858	500
NCT04350684	40	NCT04372628	900	NCT04342169	400	NCT04379479	562
NCT04330586	141	NCT04350320	102	NCT04329195	554	NCT04386616	300
NCT04361318	100	NCT04364763	252	NCT04321616	700	NCT04382651	120
NCT04361942	24	NCT04344730	550	NCT04311697	144	NCT04393246	1407
NCT04315298	400	NCT04341038	84	NCT04357730	60	NCT04390503	200
NCT04359953	1600	NCT04328272	75	NCT04367077	400	NCT04394208	50
NCT04377620	500	NCT04374487	100	NCT04360096	288	NCT04402866	159
NCT04330638	342	NCT04328480	2500	NCT04359810	105	NCT04395170	75
NCT04366739	40	NCT04350580	138	NCT03042143	75	NCT04404426	100
NCT04369742	626	NCT04323345	1000	NCT04333368	40	NCT04386694	30
NCT04363372	90	NCT04366232	50	NCT02735707	7100	NCT04395768	200
NCT04326920	80	NCT04342663	152	NCT04348695	94	NCT04402203	50
NCT04353284	114	NCT04343001	10000	NCT04347382	30	NCT04391309	300
NCT04359277	1000	NCT04356534	40	NCT04358081	444	NCT04389840	524
NCT04351763	804	NCT04344288	304	NCT04342650	210	NCT04397718	198
NCT04340544	2700	NCT04348383	120	NCT04279197	136	NCT04379076	48
NCT04366115	126	NCT04341870	27	NCT04345861	7	NCT04401475	510
NCT04366050	560	NCT04352400	256	NCT04376684	800	NCT04401475	510
NCT04341675	30	NCT04360824	170	NCT04349592	456	NCT04379271	230
NCT04329923	400	NCT04369469	270	NCT04324463	4000	NCT04390061	116
NCT04329923	400	NCT04367831	100	NCT04324463	4000	NCT04383535	333
NCT04329923	400	NCT04251767	0	NCT04371393	300	NCT04405921	200
NCT04361643	120	NCT04368923	60	NCT04351295	40	NCT04382053	120
NCT04355143	150	NCT04348513	60	NCT04363840	1080	NCT04398290	30
NCT04333628	210	NCT04257656	237	NCT04351347	300	NCT04392128	114
NCT04333628	210	NCT04338126	60	NCT03808922	250	NCT04406532	100
NCT04334382	1550	NCT04334850	194	NCT04341493	86	NCT04403646	140
NCT04357990	81	NCT04335071	100	NCT04362059	24	NCT04392531	120
NCT04335136	200	NCT04348305	1000	NCT04346446	29	NCT04385095	400
NCT04362189	110	NCT04362111	20	NCT04365582	640	NCT04390464	1167
NCT04330690	440	NCT04355364	100	NCT04363437	70	NCT04381871	110
NCT04359511	210	NCT04377503	40	NCT04325906	346	NCT04390139	30
NCT04351724	500	NCT04373460	1344	NCT04346628	120	NCT04386447	145
NCT04344444	600	NCT04343963	436	NCT04327388	409	NCT04395456	144
NCT04344236	48	NCT04349410	500	NCT04344535	500	NCT04401527	200
NCT04307693	150	NCT04354428	630	NCT04338906	334	NCT04387760	150
NCT04331899	120	NCT04351490	3140	NCT04325893	1300	NCT04393948	48
NCT04362332	950	NCT04341415	60	NCT04371367	108	NCT04387240	22
NCT04336254	20	NCT04374552	140	NCT04374539	116	NCT04390217	120
NCT04332094	276	NCT04365153	45	NCT04251871	150	NCT04397510	50
NCT04292899	6000	NCT04356937	300	NCT04361253	220	NCT04390022	24
NCT04370782	750	NCT04361032	260	NCT04322123	630	NCT04405570	44
NCT04312997	100	NCT04364009	240	NCT04363502	30	NCT04399356	100
NCT04377711	400	NCT04353271	58	NCT04322396	226	NCT04399980	60
NCT04348409	50	NCT04364737	300	NCT04346693	320	NCT04382040	50
NCT04347954	45	NCT04355728	24	NCT04344041	260	NCT04401293	308
NCT04360551	40	NCT04366245	72	NCT04321278	440	NCT04379492	120
NCT04343989	90	NCT04357457	212	NCT04345289	1500	NCT04389580	160
NCT04292730	1600	NCT04333914	273	NCT04358783	30	NCT04384445	20
NCT04358549	50	NCT04351191	400	NCT04353037	850	NCT04400929	30
NCT04345523	278	NCT04358406	60	NCT04260594	380	NCT04391179	80
NCT04346615	120	NCT04326790	180	NCT04326426	300	NCT04405739	80
NCT04244591	80	NCT04372082	480	NCT04345406	60	NCT04401150	800
NCT04329650	200	NCT04331054	436	NCT04366856	500	NCT04397497	50
NCT04331470	30	NCT04344184	200	NCT04338802	96	NCT04402957	60
NCT04320615	330	NCT04338698	500	NCT04345887	60	NCT04381377	394
NCT04372186	379	NCT04335786	651	NCT04374474	75	NCT04403100	1968
NCT04358809	480	NCT04335552	500	NCT04322773	200	NCT04385771	80
NCT04273529	100	NCT04357860	120	NCT04345419	120	NCT04381936	12000
NCT04374279	60	NCT04351516	350	NCT04347031	320	NCT04402060	66
NCT04273581	40	NCT04366063	60	NCT04350281	60	NCT04392778	30
NCT04374032	120	NCT04374019	240	NCT04343729	416	NCT04394377	600
NCT04363866	40	NCT04356495	1057	NCT04261907	160	NCT04403243	70
NCT04342221	220	NCT04346667	400	NCT04264533	140	NCT04402944	60
NCT04315896	500	NCT04354441	600	NCT04275388	426	NCT04382846	80
NCT04355767	206	NCT04347941	200	NCT04322682	6000	NCT04403555	40
NCT04338074	100	NCT04328012	4000	NCT04355052	250	NCT04395807	120
NCT04368000	60	NCT04338009	152	NCT04341727	500	NCT04404218	480
NCT04331600	400	NCT04310228	150	NCT04346927	30	NCT04380935	60
NCT04347174	40	NCT04295551	80	NCT04328467	1500	NCT04404361	358
NCT04363060	104	NCT04365985	500	NCT03852537	90	NCT04389450	140
NCT04332107	2271	NCT04273763	18	NCT04367168	174	NCT04395144	346
NCT04273646	48	NCT04369794	1000	NCT04276688	127	NCT04396067	360
NCT04349241	100	NCT04371107	64	NCT04346940	30	NCT04383717	60
NCT04363203	300	NCT04359862	50	NCT03680274	800	NCT04385186	60
NCT04252664	308	NCT04324021	54	NCT04308668	1309	NCT04382391	20
NCT04341116	144	NCT04334967	1250	NCT04346979	50	NCT04390152	40
NCT04375397	46	NCT04298060	280	NCT04401423	100	NCT04380961	270
NCT04358068	2000	NCT04332991	510	NCT04406389	186		
